# A Transgenic Platform for Testing Drugs Intended for Reversal of Cardiac Remodeling Identifies a Novel 11βHSD1 Inhibitor Rescuing Hypertrophy Independently of Re-Vascularization

**DOI:** 10.1371/journal.pone.0092869

**Published:** 2014-03-25

**Authors:** Oren Gordon, Zhiheng He, Dan Gilon, Sabine Gruener, Sherrie Pietranico-Cole, Amit Oppenheim, Eli Keshet

**Affiliations:** 1 Departments of Developmental Biology and Cancer Research, The Hebrew University–Hadassah University Hospital, Jerusalem, Israel; 2 Department of Cardiology, The Hebrew University–Hadassah University Hospital, Jerusalem, Israel; 3 Department of Metabolic and Vascular Disease, Hoffmann-La Roche Pharmaceuticals, Basel, Switzerland; Institute of Clinical Medicine, National Cheng Kung University, Taiwan

## Abstract

**Rationale:**

Rescuing adverse myocardial remodeling is an unmet clinical goal and, correspondingly, pharmacological means for its intended reversal are urgently needed.

**Objectives:**

To harness a newly-developed experimental model recapitulating progressive heart failure development for the discovery of new drugs capable of reversing adverse remodeling.

**Methods and Results:**

A VEGF-based conditional transgenic system was employed in which an induced perfusion deficit and a resultant compromised cardiac function lead to progressive remodeling and eventually heart failure. Ability of candidate drugs administered at sequential remodeling stages to reverse hypertrophy, enlarged LV size and improve cardiac function was monitored. Arguing for clinical relevance of the experimental system, clinically-used drugs operating on the Renin-Angiotensin-Aldosterone-System (RAAS), namely, the ACE inhibitor Enalapril and the direct renin inhibitor Aliskerin fully reversed remodeling. Remodeling reversal by these drugs was not accompanied by neovascularization and reached a point-of-no-return. Similarly, the PPARγ agonist Pioglitazone was proven capable of reversing all aspects of cardiac remodeling without affecting the vasculature. Extending the arsenal of remodeling-reversing drugs to pathways other than RAAS, a specific inhibitor of 11β-hydroxy-steroid dehydrogenase type 1 (11β HSD1), a key enzyme required for generating active glucocorticoids, fully rescued myocardial hypertrophy. This was associated with mitigating the hypertrophy-associated gene signature, including reversing the myosin heavy chain isoform switch but in a pattern distinguishable from that associated with neovascularization-induced reversal.

**Conclusions:**

A system was developed suitable for identifying novel remodeling-reversing drugs operating in different pathways and for gaining insights into their mechanisms of action, exemplified here by uncoupling their vascular affects.

## Introduction

Cardiac remodeling is a broad term describing the overall functional and structural changes of the myocardium in response to chronic overload or injury [1], [2], [3]. Remodeling is an adaptive process enabling the heart to withstand increased mechanical stress. Unfortunately, however, at later disease stages this beneficial adaptive process almost always becomes maladaptive and a prognostic determinant of heart failure [4].

Correspondingly, therapeutic approaches to reverse maladaptive remodeling are currently considered a prime clinical goal. In principle, intended reversal can be attained through two different approaches: correcting its underlying cause, e.g. by restoring perfusion to the ischemic myocardium or, alternatively, by a direct pharmacological intervention without necessarily rectifying the underlying cause [5].

On the basis of findings that the renin angiotensin aldosterone system (RAAS) plays a major role in the remodeling pathogenesis [6] RAAS inhibitors were developed and proven useful in alleviating clinical symptoms associated with adverse remodeling, including using Angiotensin converting enzyme inhibitors (ACEIs), Angiotensin receptor blockers (ARBs) and direct renin inhibitors (DRIs) [7]. Unfortunately, however, in most cases currently used drugs come up short in preventing further disease progression [8] thus begging for introduction of new and more efficient drugs. This might require expanding the drug arsenal to include not only drugs belonging to the RAAS family but also drugs affecting other pathways, e.g., cardiac metabolism. The peroxisome proliferator-activated receptor family (PPARα, β/δ, γ) of nuclear receptor transcription factors is an important regulator of cardiac metabolism and was harnessed for targeting cardiac metabolism [9]. A PPARγ agonist was indeed capable of attenuating left ventricular remodeling and failure in a coronary ligation model [10]. Yet, reversing remodeling in heart failure remains a major challenge and new opportunities continue to be sought (for a recent review see #5).

Suitable animal models of heart failure have been instrumental for testing the potential utility of remodeling-reversing drugs and elucidating their mode of action [11], [12], [13]. In these model systems myocardial insults are inflicted using either a surgical procedure (e.g. ligating the left coronary artery (LAD) [14]) or a pharmacological intervention (e.g., administrating the β1 adrenergic receptor agonist isoproterenol [13]). To avoid confounding factors associated with these manipulations, genetic systems for inducing cardiac hypertrophy were developed, including transgenic mice expressing an activated Akt1 [15] gene or transgenic rats over-expressing the renin gene [11]. Yet, a number of large clinical trials prompted by encouraging preclinical studies obtained with the aid of these animal models did not meet expectations [16]. This likely reflects the fact that different insults converging on the common pathway of myocardial remodeling are accompanied by additional processes that might differ between different pathologies not accurately reproduced by the particular animal model. Hence, a complementary animal model displaying gradual progression of ischemic heart disease (IHD) to heart failure and also better amenable to experimental manipulations is highly desired. To this end, we have developed a transgenic system based on conditional (and reversible) blockade of VEGF signaling for the purpose of generating myocardial perfusion deficits of escalating magnitudes. This manipulation leads to development of IHD closely resembling dilated ischemic cardiomyopathy and stepwise development of all hallmarks of cardiac remodeling eventually culminating in heart failure [17]. The system is particularly suitable for studying remodeling reversal as evidenced by complete reversal following VEGF-mediated myocardial re-vascularization [18]. Moreover, because the disruption of coordinated cardiac hypertrophy and angiogenesis contribute to transition to heart failure [15], the system provides a unique opportunity to uncouple the beneficial activity of the tested drug on reversing myocardial hypertrophy from its vascular effect. Another advantage of the model for studying remodeling and its reversal is its slow, stepwise development thus allowing attempted reversal at different progressive stages. Indeed, we have previously shown that there is a point-of-no-return beyond which remodeling is no longer reversible via re-vascularization [18] and the question remains whether a similar restriction point also exists for drug-induced reversal.

Here we provide a proof of principle for the general utility of our experimental platform for the discovery of remodeling-reversing drugs by first corroborating the efficacy of known drugs affecting the RAAS pathway, namely, the ACE inhibitor Enalapril and the direct renin inhibitor Aliskiren, as well as of the PPARγ agonist Pioglitazone. Exemplifying the utility of the experimental system for the discovery of new agents capable of reversing cardiac hypertrophy are finding reported here employing a specific 11β-hydroxysteroid dehydrogenase type 1 (11β HSD1) inhibitor. 11β HSD1 catalyses intracellular regeneration of active glucocorticoids (cortisol, corticosterone) from inert 11-keto forms in target tissues, amplifying local action [19]. Transgenic mice overexpressing 11β-HSD1 selectively in adipose tissue faithfully recapitulate metabolic syndrome. Conversely, 11β-HSD1 knockout mice have a ‘cardioprotective’ phenotype [20], [21]. Here, we show that a newly developed inhibitor of 11β-HSD1 (Hoffmann-La Roche) completely reverses cardiac hypertrophy even in the face of persisting compromised perfusion and a compromised cardiac function.

## Materials and Methods

### Transgenic mice and conditional modulations of VEGF signaling

A bitransgenic system for organ-specific, tetracycline-regulated transgene expression was used. Heart-specific induction was achieved by using a transgenic driver line in which tTA expression is driven by a myosin heavy chain (MHC) heart-specific promoter [22]. The tet–sVEGF-R1 transgenic line encodes a tetracycline-inducible protein composed of an IgG1–Fc tail fused to the extracellular domain of VEGF-R1 (corresponding to amino acid residues 1–631 of human VEGF-R1 containing the ligand-binding domain but lacking the transmembrane and cytoplasmic domains). Induction of sVEGF-R1 in double-transgenic mice was accomplished by tetracycline withdrawal and the termination of sVEGF-R1 by tetracycline addition (0.5 mg/ml tetracycline and 3% sucrose in the drinking water). Animals were sacrificed using 200 mg/kg pentobarbital IP, followed by removal of the heart. All animal experiments conform to the Guide for the Care and Use of Laboratory Animals published by the US National Institutes of Health and were done under approval of the Hebrew University ethics committee, approval number- MD-10127064.

### Drugs

All drugs were administered for 3 weeks. Enalapril (Sigma) was administered in drinking water (50 mg/L). Aliskiren (Novartis) was administered using 2004 Alzet miniosmotic pumps by Durect Corp. (50 mg/kg/d). Pioglitazone (ChemPacific) and the 11β-HSD1 inhibitor, Roche Compound (Cpd)-A (Hoffmann-La Roche) were administered in a food admix (15 mg/kg and 60 mg/kg, respectivly). Isoproterenol (Sigma) was administered to 5–6 weeks old C57BL/6JOLaHsd male mice S.C. (15 mg/kg/day) for 5 weeks.

### Immunohistochemistry

4% paraformaldehyde-fixed paraffin-embedded specimens were used. Sections (5 um) were rehydrated and antigen was retrieved by 0.1% pronase (Sigma) for 20 min at room temperature. Endogenous peroxidase activity was quenched with hydrogen peroxide 3% in PBS for 15 minutes at room temperature. Sections were blocked by incubation in 1% BSA (Amresco) in PBS at room temperature followed by overnight incubation with primary antibody: mouse monoclonal anti-myosin (M8421: skeletal, slow; 1:2000, Sigma). Endothelial cells were visualized by using *Bandeiraea simplicifolia* isolectin B4 staining.

### RNA analysis

For real-time PCR analysis, cDNA was generated from 1 μg of total RNA, extracted from the apex of the left ventricle, by using Verso cDNA kit (Thermo scientific) and the respective primers. Samples were normalized according to L19 mRNA levels by SYBR green real-time PCR (StepOnePlus™ Real-Time PCR System, Applied Biosystems).

### Echocardiography

Transthoracic echocardiography was performed on shallow anesthetized mice (ketamine and xylazine 200 mg/kg IM and 10 mg/kg IP ). For more details please refer to the supplemental information. The high-resolution ultrasound imaging system Vevo 770 (VisualSonics, Toronto, Canada) was used to perform two-dimensional (B-mode) and motion-mode (M-mode) imaging using a mechanical transducer (RMV707B) synchronized to the electrocardiographic signal. The transducer had a central frequency of 40 MHz, a focal length of 6 mm, a frame rate of 30 Hz, and an 8×8 mm field of view with spatial resolution of 30 μm. A 2D-directed M-mode image of the left ventricular short axis was taken just at the level of the papillary muscles, and measurements were performed in triplicate by using the leading-edge convention for myocardial borders, as defined by the American Society of Echocardiography [23]. The following parameters were measured: left ventricular end-diastolic diameter (LVEDD); left ventricular end-systolic diameter (LVESD) and heart rate. The percentage fractional shortening (%SF) was calculated as %SF  =  [(LVEDD - LVESD)/(LVEDD)] × 100.

### Statistical analysis

Numerical data are the mean ± standard error. Statistical significance was determined by using student's t-test.

## Results

### A transgenic system for generating a volume overload-driven cardiac remodeling

**Figure 1:**
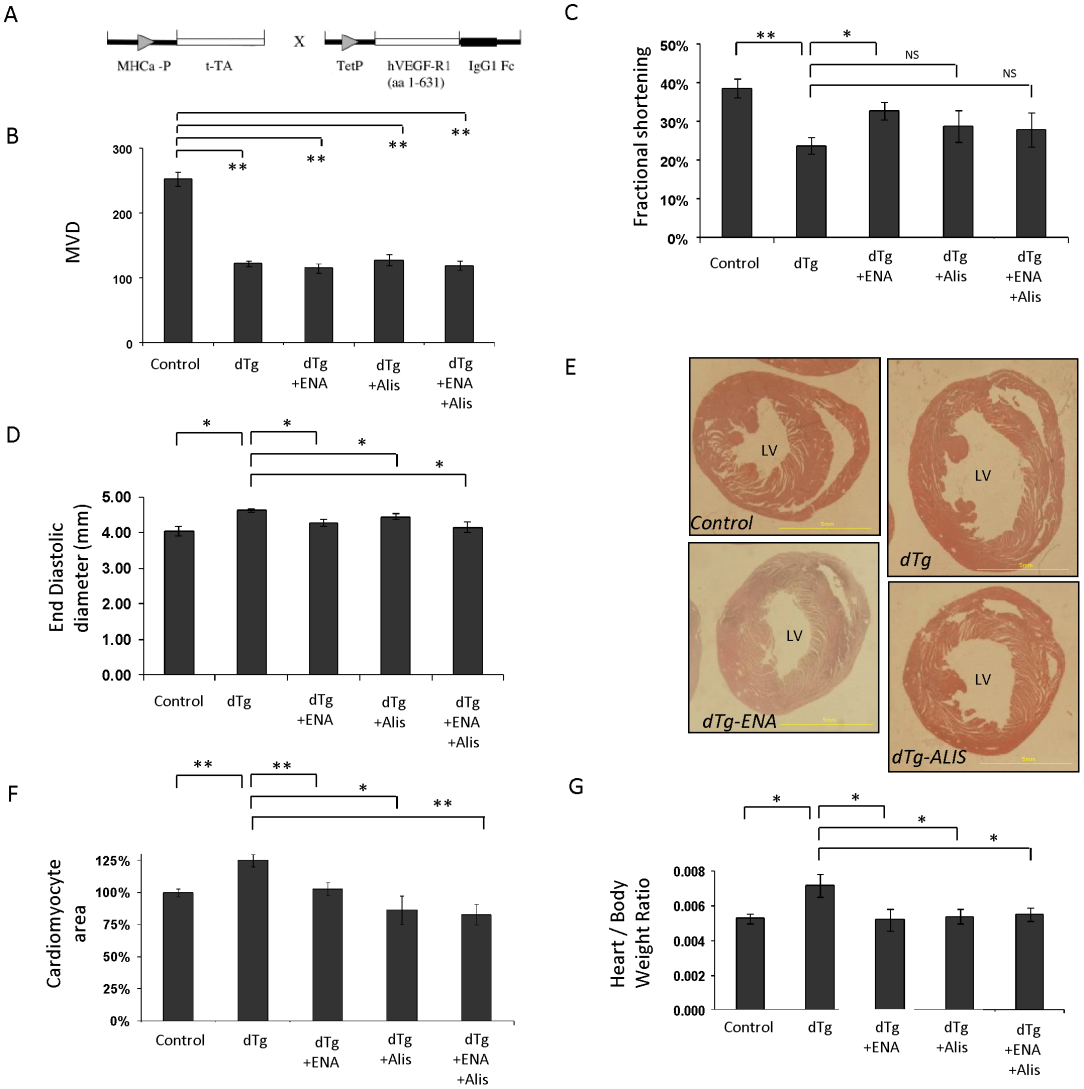
A novel experimental system for testing remodeling reversing drugs shows RAAS inhibition reverses remodeling independently of improving perfusion. (*A*) Schematic representation of the transgenic lines used in the bi-transgenic inducible system. (*B*) Quantification of micro-vessels density (MVD), expressed as the number of endothelial cell-specific lectin-positive capillaries per high-power field (HPF). MVD was determined after 12 weeks of sVEGF-R1 induction during the last 3 weeks of which some of the dTg mice were treated with Enalapril (ENA) or Aliskiren (Alis) or both. N =  6–16 mice per treatment group and 3 HPFs were measured for each mouse. (*C*) Fractional shortening measured by M-mode Echocardiography in mice treated as in B. N = 5 mice. (*D*) End diastolic LV diameter measured by M-mode Echocardiography. (*E*) Representative axial sections at the level of the papillary muscle showing drug-induced reduction of LV size. (*F*) Cardiomyocyte area was measured from H&E-stained sections using Image J software and was standardized to littermate controls. N = 6-12 mice and 25-30 cardiomyocytes were analyzed for each section. (*G*) Heart to body weight ratio in the same mice shown in F. * *P*<0.05, ** *P*<0.001, NS  =  not statistically significant.

Previously, we developed a transgenic system where VEGF blockade led to microvascular insufficiency and resulted in development of an IHD-like phenotype [17], [18]. Briefly, because VEGF is indispensable for adjusting the coronary microvasculature to match dynamic changes in oxygen supply and demand, inhibition of myocardial VEGF signaling generates a perfusion deficit the magnitude of which depends on the duration of VEGF blockade [17]. A tetracycline-regulated VEGF decoy receptor (sVEGF-R1) is conditionally induced in the heart (pending on the absence or presence of doxycycline in the drinking water) using a cardiomyocyte-specific promoter driving expression of a trans-activator protein (see Fig 1A for a scheme and "Methods" for experimental details). In agreement with previous studies [17], [18], when switched ‘on’ at an early post-natal age and maintained in the ‘on’ mode for 9 weeks, sVEGF-R1production in double transgenic (dTg) mice causes microvascular density (MVD) reduction and a markedly reduced as monitored by echocardiography and evidenced by a reduced fractional shortening (left lanes in Figs 1B and 1C). Volume overload resulting from a compromised cardiac function is likely the cause of apparent remodeling manifested by LV enlargement (left lanes in Figs 1D and 1E) and myocardial hypertrophy (left lanes in Figs 1F and 1G).

### Harnessing the transgenic system for measuring efficacy of remodeling reversing drugs - feasibility testing using ACE inhibitors

To prove the general utility of our experimental platform to identify pharmaceuticals capable of reversing remodeling, we first tested the performance of a drug already in clinical use for this purpose, namely, the ACE inhibitor Enalapril. To this end we induced LV remodeling as described above. Enalapril was then added and heart retrieved for analysis 3 additional weeks later. Importantly, Enalapril action was tested under condition of ongoing VEGF blockade as indeed reflected by the persistence of the MVD deficit (Fig.1B). Yet, Enalapril treatment led to marked reduction of LV diameter (Figs. 1D&E), fully reversed myocardial hypertrophy (Fig.1F) and partially rectified the compromised contractile function evidenced by improved fractional shortening (Fig.1C).

### Reversing cardiac remodeling by the direct renin inhibitor Aliskiren

Direct renin inhibitors have been developed in an attempt to modulate the RAAS more effectively, reasoning that they may provide additional protection over other RAAS inhibitors like ACEIs or ARBs. Aliskiren, a direct renin inhibitor recently introduced (Novartis) as an anti-hypertension drug, was shown to also reverse remodeling in a coronary ligation-induced MI model independently of its effect on blood pressure [24]. To determine whether Aliskiren will have a beneficial effect in our model, we administered Aliskiren to animals in which myocardial remodeling has been pre-induced using an identical protocol to that described above for Enalapril. Results showed that Aliskiren was as effective as Enalapril in reversing remodeling (Fig.1). Interestingly, the combined effect of Enalapril and Aliskiren failed to show an additive effect over that shown for each drug administered alone (Fig.1). Results thus demonstrate the utility of this experimental system for screening new candidate drugs intended for reversing remodeling.

### Advanced stages of remodeling are refractory to drug-induced reversal

**Figure 2:**
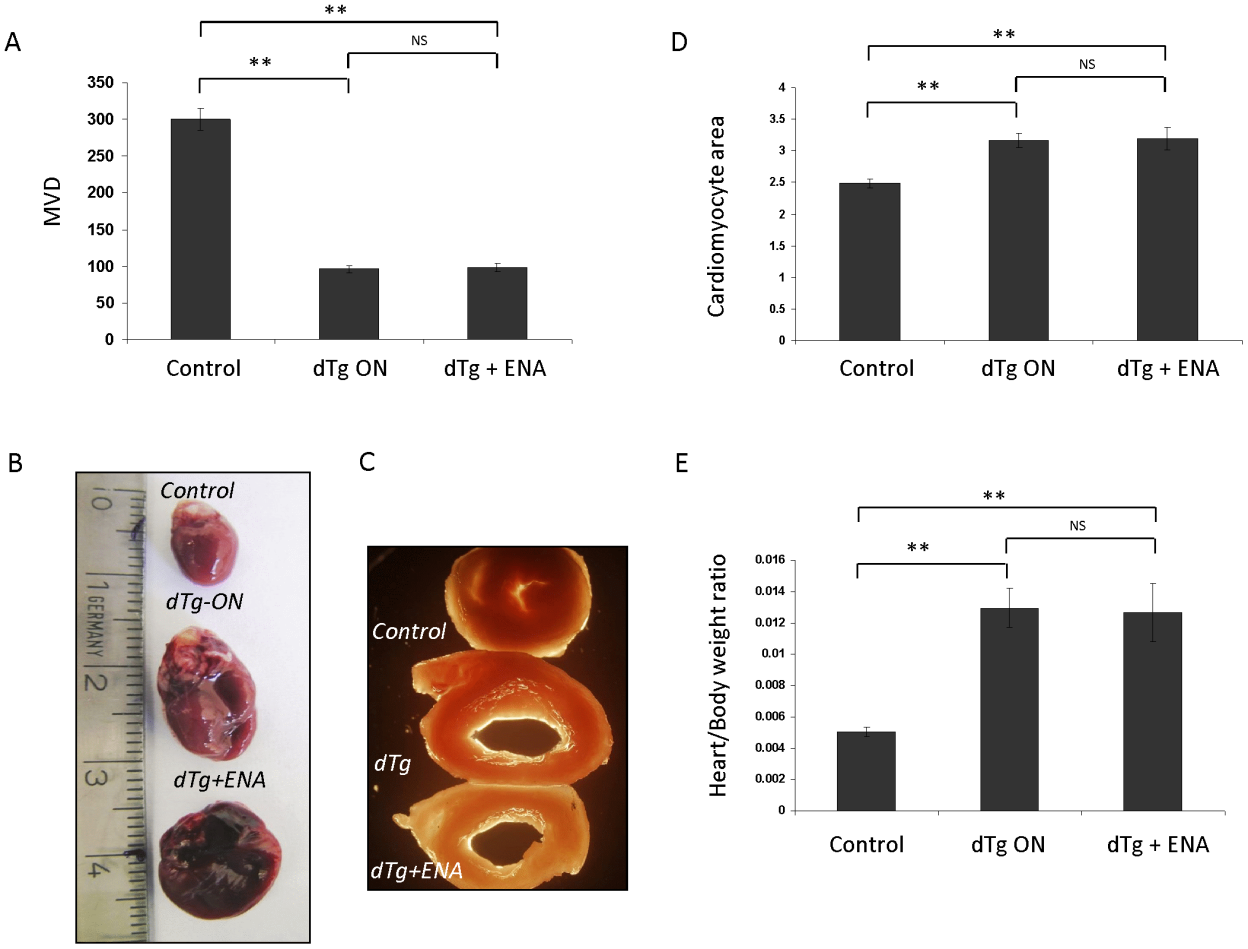
A Point-of-no-return for pharmacological reversal of heart remodeling. (*A*) MVD was determined after 35weeks of sVEGF-R1 induction during the last 3 weeks of which some of the dTg mice were treated with Enalapril (ENA). N =  3 mice per treatment group and 3 HPFs were measured for each mouse. (*B*) Gross examination of cardiac size and comparison between control (upper), dTg at 35 week induction (middle) and dTg after 32 weeks induction and 3 weeks of Enalapril treatment (lower). (*C*) Gross sections at the level of the papillary muscles of control and dTg hearts with and without 3 weeks treatment, as indicated. (*D*) Cardiomyocyte area in control and dTg hearts with or without the indicated treatment. N = 3 mice and 25-30 cardiomyocytes were analyzed for each section. (*E*) Heart to body weight ratio in the same mice shown in D.

A major concern in intended rescue of adverse remodeling is that it may reach a refractory stage where it is no longer responsive to remodeling-reversing drugs. We have recently used the system to show that restoring adequate perfusion to the ischemic myocardium via VEGF-induced neovascularization can rescue remodeling at early stages of IHD but fails to do so at an advanced stage distinguished by critical levels of fibrosis [18]. To determine whether a similar point-of-no-return also exists for drug-induced reversal, we have attempted reversal by Enalapril 32 weeks from the onset of VEGF blockade, i.e., a time point during progressive remodeling beyond the point-of-no return for re-vascularization-induced reversal [18]. As shown in Fig.2 and contrary to the earlier time point of 9 weeks post-induction where remodeling was still reversible (Fig.1), Enalapril failed to do so by 32 days post-induction as evidenced by no decrease in LV size and no resolution of hypertrophy. This was further validated through gene expression analysis of remodeling-associated genes (Fig S1 and see below).

### Reversing cardiac remodeling by the PPARγ agonist Pioglitazone in an angiogenesis-independent manner

The PPAR system play an important role in regulating myocardial metabolism in health and disease [25]. Its role in heart remodeling is evidenced by findings that mice with a cardiomyocyte-specific knockout of PPARγ develop cardiac hypertrophy [26]. Correspondingly, modulating the PPAR pathway was recognized as an attractive therapeutic avenue for treating cardiac remodeling and Pioglitazone, a PPARγ agonist, was shown to attenuate cardiac remodeling and heart failure after experimental myocardial infarction (MI) [10].

**Figure 3:**
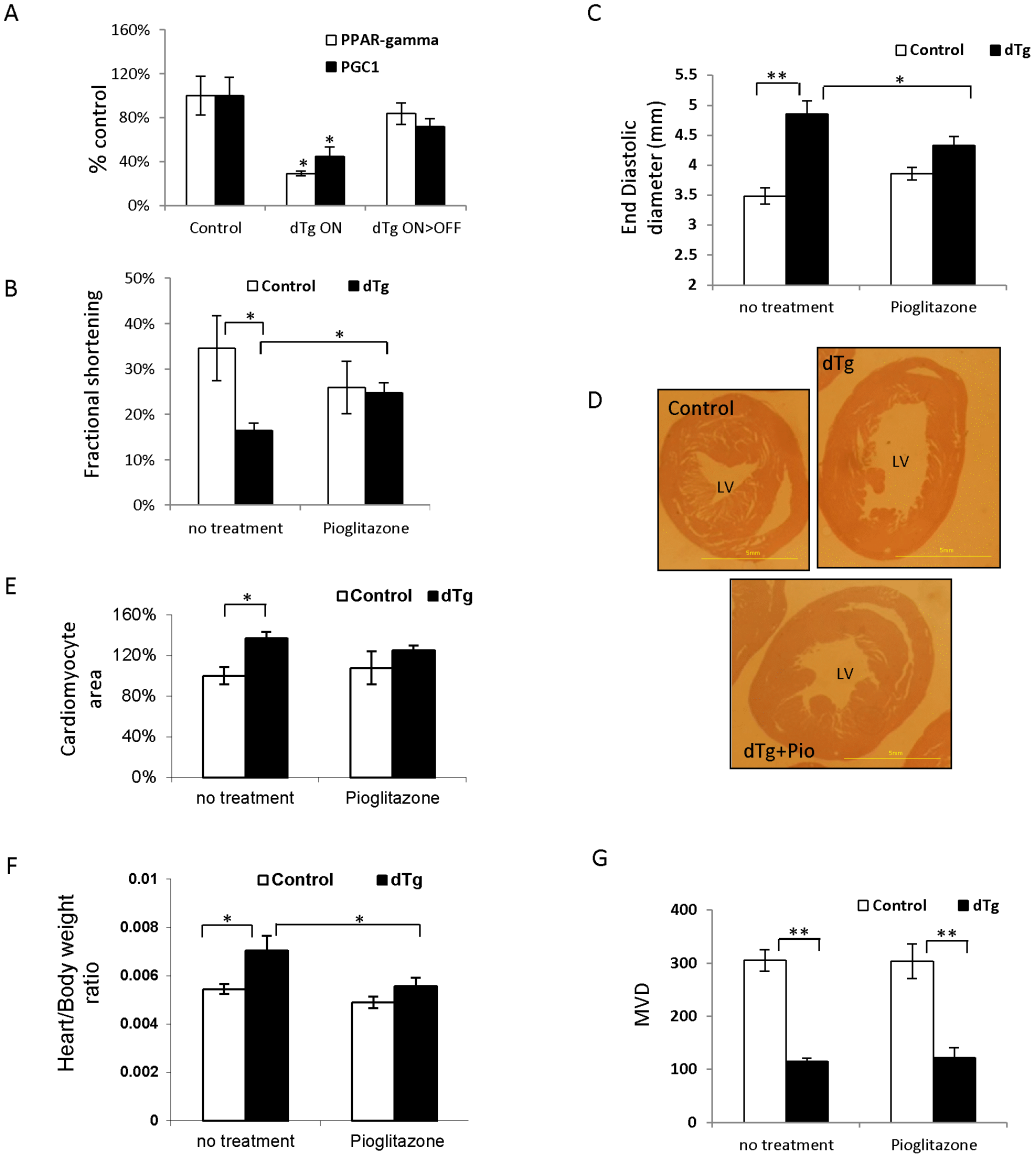
Pioglitazone, a PPARγ agonist, reverses cardiac remodeling without effecting vascular density. (*A*) Real time PCR results for PPARγ and PGC1α after 12 weeks of sVEGF-R1 induction (dTg-ON), during the last 3 weeks of which in some of the dTg mice, sVEGF-R1 was de-induced (dTg-ON>OFF). (*B*) Fractional shortening was determined after 12 weeks of sVEGF-R1 induction during the last 3 weeks of which some of the dTg mice were treated with Pioglitazone. N = 4–6 mice. (*C*) End diastolic LV diameter. (*D*) Representative axial sections at the level of the papillary muscle showing drug-induced reduction of LV size. (*E*) Cardiomyocyte area standardized to littermate controls. N = 4–6 mice and 25–30 cardiomyocytes were analyzed for each section. (*F*) Heart to body weight ratio in the same mice shown in E. (*G*) MVD. N =  4–6 mice per treatment group and 3 HPFs were measured for each mouse. * *P*<0.05, ** *P*<0.001, NS  =  not statistically significant.

To determine whether the metabolic stress incurred in our experimental model also leads to changes in expression of genes of the PPAR system, we first examined the expression levels of PPARγ and PGC1α, a co-activator of the PPARs during heart remodeling and following its reversal. As shown in Fig.3A, both genes were indeed strongly downregulated in the remodeled myocardium and returned to normal expression level upon alleviating the perfusion deficit and the resolution of myocardial remodeling. To examine whether, similarly to its activity in the coronary ligation model, Pioglitazone can reverse remodeling and improve cardiac function in our model as well, we performed similar experiments to those described above for inhibitors of the RAAS pathway. Pioglitazone was found to improve cardiac function evidenced by increased fractional shortening (Fig. 3B) and to resolve cardiac remodeling evidenced by reducing LV size (Fig 3C and D) and reversing myocardial hypertrophy (Figs 3E and F). Notably, these beneficial actions of Pioglitazone took place without improving the vascular deficit (Fig.3G). Note that in accordance to previous reports, Pioglitazone affects cardiac function and end-diastolic diameter in control mice (Fig 3B and C), these differences where statistically insignificant in this study.

### Reversing cardiac hypertrophy by 11β-hydroxysteroid dehydrogenase type 1 (11β HSD1) inhibition

11β-HSD1 catalyzes intracellular regeneration of active glucocorticoids (cortisol, corticosterone) from inert 11-keto forms in target tissues thus amplifying local action on the glucocorticoid receptor and possibly the mineralocorticoid receptor [19]. The possible involvement of 11β-HSD1 in cardiac remodeling was prompted by findings that 11β-HSD1 gene expression was increased in experimentally induced hypertrophy, *in vitro* [27] and findings that 11β-HSD1 knockout mice show enhanced angiogenesis and improved heart function in an MI model [20], [21].

**Figure 4:**
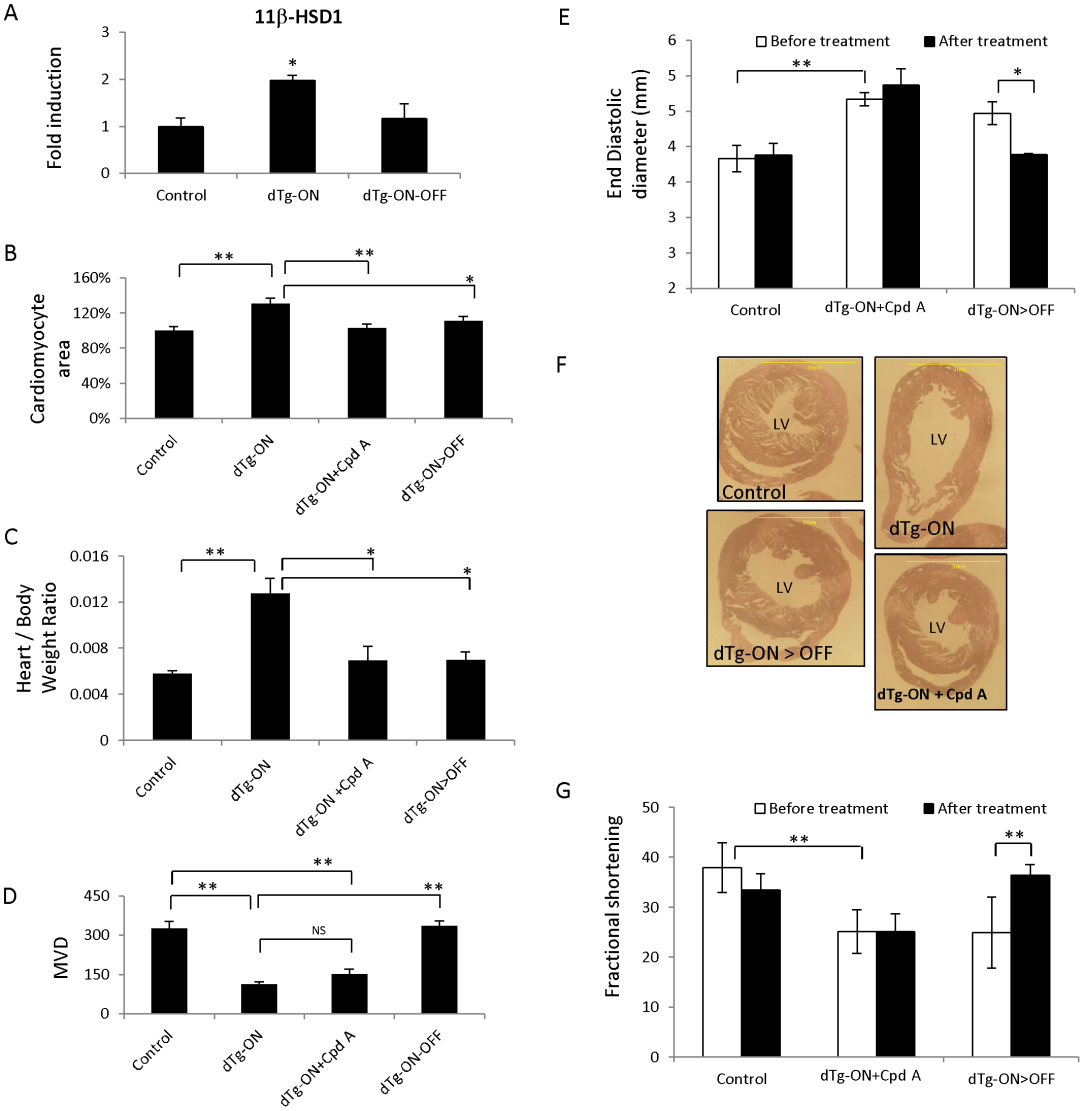
11β-HSD1 inhibiton reverses cardiac hypertrophy without effecting vascular density and cardiac function. (*A*) Real time PCR results for 11β-HSD1 after 12 weeks of sVEGF-R1 induction (dTg-ON), during the last 3 weeks of which in some of the dTg mice, sVEGF-R1 was de-induced (dTg-ON>OFF). (*B*) Cardiomyocyte area standardized to littermate controls, was determined after 12 weeks of sVEGF-R1 induction during the last 3 weeks of which some of the dTg mice were treated with 11β-HSD1 inhibitor (Roche Cpd A) or de-induced (dTg-ON>OFF). N = 4–5 mice and 25–30 cardiomyocytes were analyzed for each section. (*C*) Heart to body weight ratio in the same mice shown in B (*D*) MVD. N =  4–5 mice per treatment group and 3 HPFs were measured for each mouse. (*E*) End diastolic LV diameter. (*F*) Representative axial sections at the level of the papillary muscle. (*G*) Fractional shortening measured in mice treated as in B. N = 4–5 mice. * *P*<0.05, ** *P*<0.001, NS  =  not statistically significant.

To examine in our model whether the level of 11β-HSD1 changes in accordance with the remodeling status, we compared relative levels of expression of the endogenous gene in control and remodeled myocardium, as well as following neovascularization-induced reversal (Fig.4A). Result showed that remodeling is associated with 2-fold increase in 11β-HSD1 expression and that 11β-HSD1 expression returns to normal levels following restoration of normal perfusion via VEGF-mediated neovascularization, thus prompting the proposition that elevated 11β-might is required to maintain the remodeling state and hence its inhibition might lead to reversion.

To test this proposition, we attempted remodeling reversal by a novel 11β-HSD1 inhibitor, Roche Compound A (Hoffmann-La Roche)(Cpd A for short). Preparatory experiments have exhibited up to 90% inhibition of 11β-HSD1 enzymatic activity when used at the same dose as the dose applied in the experiments described below (Fig.S2). Remodeling was induced as described above for the testing of RAAS inhibitors and a PPARγ agonist (VEGF blockade for 9 weeks) followed by 11β-HSD1 inhibitor administration for 3 weeks.

Usually, progressive deterioration towards heart failure in our system is accompanied by progressive weight loss which was prevented by the 11β-HSD1inhibitor: mice re-gained normal weight comparable to that of littermate controls or following neovascularization-induced reversal (Fig.S3).

Remarkably, the 11β-HSD1 inhibitor fully rescued myocardial hypertrophy and was as efficient in this regard as hypertrophy reversal via neovascularization (Figs. 4B and C). Noteworthy, 11β-HSD1 inhibition acted to reverse hypertrophy in face of a remaining MVD shortage (Fig.4D) and its sequela. 11β-HSD1 inhibition did not affect cardiac function and end-diastolic diameter (Fig.4E-G).

**Figure 5:**
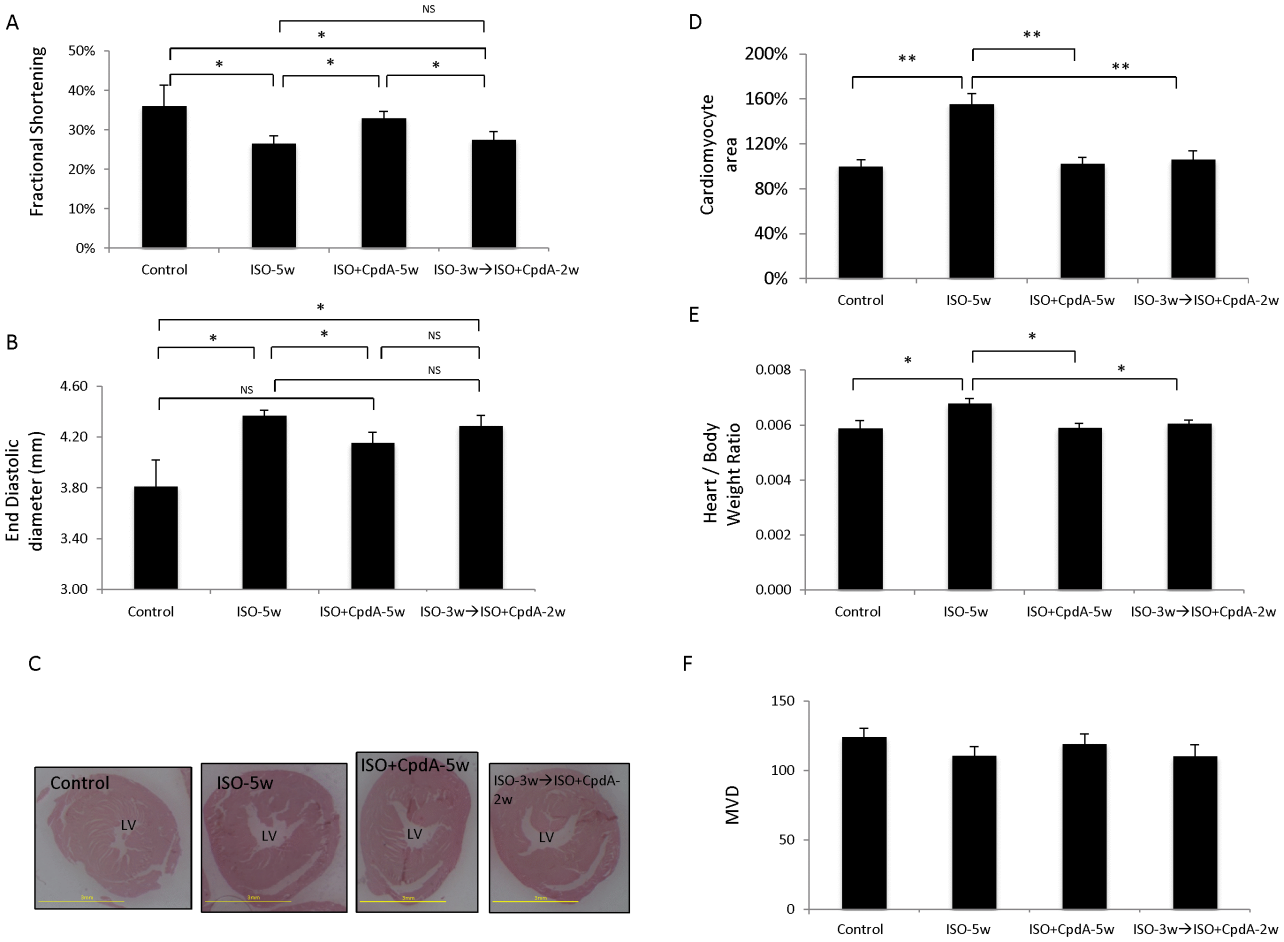
Pharmacological inhibition of 11β HSD1 reverses cardiac hypertrophy in a model of Isoproterenol-induced heart failure. (*A*) Fractional shortening measured in mice treated for 5 weeks with Isoproterenol(ISO-5w) alone, Isoproterenol together with 11β-HSD1 inhibitor (ISO+CpdA-5w) or treated with Isoproterenol alone for 3 weeks and by Isoproterenol and 11β-HSD1 inhibitor for an additional 2 weeks (ISO-3w→ISO+CpdA-2w). N =  5–7 mice per treatment group. (*B*) End diastolic LV diameter in the same mice as in A. (*C*) Representative axial sections at the level of the papillary muscle. (*D*) Cardiomyocyte area standardized to littermate controls. N = 5–7 mice and 25–30 cardiomyocytes were analyzed for each section. (*E*) Heart to body weight ratio. (*F*) MVD. N =  5–7 mice per treatment group and 3 HPFs were measured for each mouse. * *P*<0.05, ** *P*<0.001, NS  =  not statistically significant.

To validate the general utility of the 11β-HSD1 inhibitor in reversing hypertrophy, we examined its performance in an independent model of heart failure, namely the widely-used model of Isoproterenol-induced cardiac hypertrophy and heart failure [11], [13], [28]. Isoproterenol (ISO), a synthetic catecholamine and a sympathomimetic β-adrenergic receptor agonist, causes severe stress to the myocardium resulting in an infarct-like necrosis of heart muscle. Mice were treated with ISO for 5 weeks resulting in decreased cardiac function (Fig 5A, left lanes), increased cardiac size (Fig 5B and C, left lanes) and significant cardiac hypertrophy (Fig 5D and E, left lanes). When administered together with Isoproterenol (a ‘preventive mode’), the 11β-HSD1 inhibitor mitigated the effects of Isoproterenol on cardiac function and size and completely prevented cardiac hypertrophy (Fig 5). When added after 3 weeks of Isoproterenol treatment, i.e., after cardiac function has already been compromised (data not shown), the 11β-HSD1 inhibitor was still capable of completely reversing hypertrophy (Fig 5D and E) even though there was no significant improvement of cardiac function (Fig 5A) or LV end-diastolic diameter (Fig 5B). Likewise, the 11β-HSD1 inhibitor acted to reverse the hypertrophy-associated gene signature in a similar manner to that observed for neovascularization-induced reversal (Fig S3 and see below). Thus, 11β-HSD1 inhibition was proven capable of reversing pathological hypertrophy in two independent experimental models initiated by two different insults.

### Rescue of hypertrophy is associated with reversing the hypertrophic gene signature, including of the Myosin heavy chain isoform shift

Cardiac remodeling is associated with extensive alterations in myocardial gene expression and correspondingly, its reversal is likely to be reflected in a return to normal level of expression of genes mediating these pathways. Considering the complex nature of heart remodeling and the different sub-processes involved, different compounds employed for intended reversal and operating on different biochemical pathways are likely to produce differential responses with regard to changes in gene expression. Opportunities provided by the VEGF-based conditional transgenic system to monitor remodeling reversal induced by different treatment modalities (neovascularization vs. pharmacologic intervention) and different drugs has provided a suitable access to this issue.

**Figure 6:**
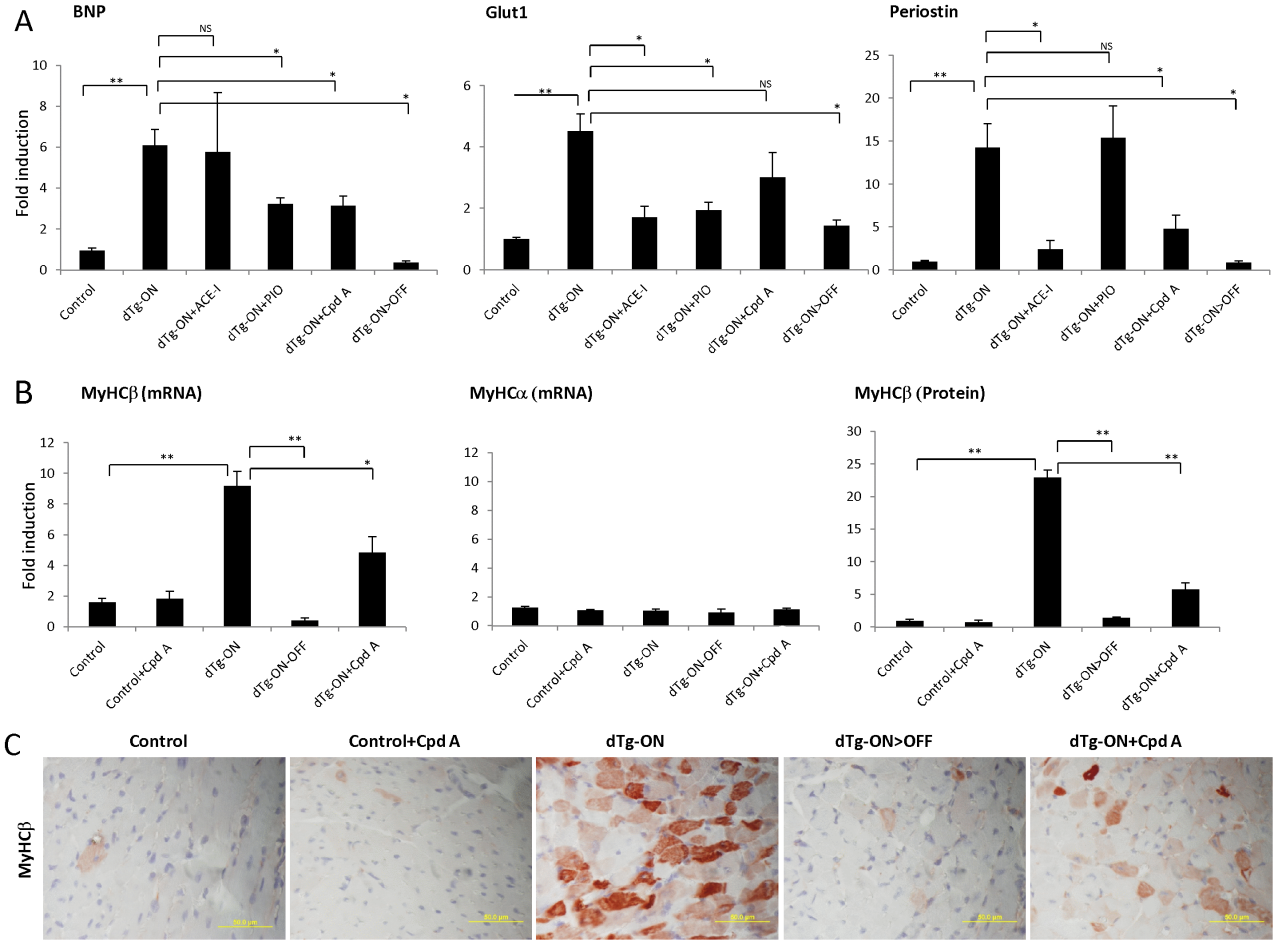
Rescue of hypertrophy is associated with reversing the hypertrophic gene signature, including of the Myosin heavy chain isoform shift. (*A*) Real time PCR results of selected genes in control and dTg (dTg ON) mice after 12 weeks of sVEGF-R1 induction, with or without treatment in the last 3 weeks. Treatment groups included either 11βHSD1 inhibitor (Roche Cpd A), Enalapril (ACE-I), Pioglitazone (PIO) or VEGF-driven neo-vascularization by sVEGF-R1 withdrawal (dTg ON>OFF). Results are normalized relative to litter-mate controls (designated as 1). N = 4-6 mice. Genes examined: Brain naturetic peptide (BNP). Glucose transporter 1 (Glut1) and Periostin. See text for further elaboration. (*B*) Real time PCR results of MyHCβ and MyHCα in control and dTg (dTg ON) mice after 12 weeks of sVEGF-R1 induction, with or without treatment in the last 3 weeks. N = 4–6 mice. Quantification (right diagram in *B*) and representative images (C) of immunohistochemistry for MyHCβ in the same mice as in B, depicting MyHCβ protein level elevation in dTg mice and rescue by both treatments. Scale bars: 50 µm. * *P*<0.05, ** *P*<0.001, NS  =  not statistically significant.

Among the different pathways implicated in cardiac remodeling outstanding are neuro-hormonal activation, multiple metabolic adaptations, and activation of fibrosis-promoting pathways (e.g. components of the TGF-β pathway) [3]. Here we examined representative genes of these respective pathways, namely BNP, a clinically used neuro-hormonal marker of heart failure induced by wall stress, the glucose transporter Glut1, an hypoxia-induced gene upregulated in cardiac ischemia^22, 23^ and Periostin, a matricellular protein promoting matrix organization following cardiac injury under the regulation of Angiotensin II and TGFβ [29], [30]. As expected. All three genes were markedly (5 to15-fold) upregulated during heart remodeling (lanes marked as ‘dTg ON’ in Fig.6A). Neovascularization-induced reversal of remodeling led to downregulation of all three genes back to their normal low level of expression (lanes marked as ‘dTg ON>OFF’ in Fig.6A). In contrast, drug-induced reversal was effective in downregulating some but not all genes. More interestingly, drugs operating on different pathways exhibited a differential response towards the different target genes. Thus, RAAS inhibition by Enalapril led to reduction of Glut1 and Periostin but not of BNP, the PPARγ agonist Pioglitazone led to a reduction of Glut1 and BNP but not of Periostin levels and the 11β-HSD1 inhibitor led to reduction of BNP and Periostin, but not of Glut1 levels (Fig.6A). Analysis of 11β-HSD1 inhibition in the Isoproterenol-induced model yielded similar results by restoring normal levels of expression of ANP (another marker of cardiac hypertrophy and periostin but not of Glut1 (Fig.S4). These finding thus suggest different points of entry for intended reversal of myocardial hypertrophy.

A gene expression change of particular functional significance taking place in hypertrophied cardiomyocytes is a myosin isoform switch from the fast-contracting myosin heavy chain MyHCα to the less energy consuming but slower contracting MyHCβ [31]. We, therefore, wished to determine whether the novel hypertrophy –reversing drug described herein acting through 11β-HSD1 inhibition is also capable of reversing the MyHC isoform shift. As shown in figure 6B, expression of MyHCβ was markedly upregulated in remodeled hearts (dTg-ON) while MyHCα was unchanged. Neovascularization-induced reversal of hypertrophy (dTg ON>OFF) resulted in downregulation of MyHCβ expression back to control levels and 11β-HSD1 inhibition had a similar though smaller effect. These results were corroborated using MyHCβ-specific immune-staining (Fig.6C) thus demonstrating the ability of 11β-HSD1 inhibition to alter myofilament composition within cardiomyocytes as a component of its overall activity in hypertrophy reversal.

## Discussion

Meeting the urgent clinical need for means to reverse adverse cardiac remodeling has been hampered by the fact that commonly used animal models employed for testing candidate drugs are confounded by the ramifications of surgical interventions and other confounding factors like massive cell death and inflammation usually associated with these models. More importantly, currently used models do not faithfully recapitulate the slow and progressive nature of heart failure development. These confounding factors are filtered-out in the transgenic system we have developed and, importantly, LV remodeling develops in this system in a slow and progressive manner. These properties provide a unique opportunity to not only examine the efficacy of candidate drugs, in general, but also to compare its potential utility when applied at consecutive stages of progressive remodeling. This is exemplified here by showing that Enalapril is effective in reversing LV remodeling when administered at an early stage but not when administered at a late stage. This finding suggests that there is a restricted time window for an attempted reversal of myocardial remodeling, similarly to the point-of-no-return we have recently demonstrated for neovascularization-induced reversal [18]. A future challenge to be addressed using this experimental platform is to identify drugs effective at the latest stage of remodeling.

An issue to be considered is the potential added value in using two or more drugs together. Here we examined the combined action of Enalapril and Aliskiren, drugs inhibiting two different components of the RAAS pathway, but did not observe any added value over the solitary beneficial effect of each drug. This result might explain the failure of Aliskiren to reveres remodeling after MI in a clinical trial in which Aliskiren was added to standard therapy [32]. Perhaps more advantageous will be an approach of a combined use of drugs operating on different remodeling-promoting pathways. This proposition is supported by our initial gene expression analysis where a successful remodeling reversal induced by drugs operating on three different pathways, namely the RAAS pathway, cardiac metabolism and glucocorticoid biogenesis, was in all cases associated with gene expression changes reflecting the return to a normal state, however, different upregulated genes signifying remodeling where downregulated in each case. A more comprehensive expression analysis may provide better rationales for using particular drug combinations with an additive (or even synergistic) value.

Because remodeling is a natural response secondary to a compromised cardiac output, treatment modalities intended for reversing adverse remodeling can either rectify the primary cause (e.g. perfusion insufficiency) or, alternatively, directly target the remodeling process without necessarily correcting its underlying cause. For RAAS inhibitors, it has been argued that at least some of its effects on heart remodeling and its reversal are mediated by promoting angiogenesis [33]. Here we took advantage of our ability to fully block angiogenesis to demonstrate that contrary to this report complete reversal by Enalapril or Aliskiren could be achieved in the complete absence of angiogenesis. In fact, the beneficial reversal effect exerted by all four drugs examined in this study was achieved without any angiogenesis response, arguing that drug-induced reversal, at least by these drugs targeting these pathways is feasible even without rectifying the perfusion deficit.

11β-HSD1 inhibition was proved by this study as a novel approach to effectively reverse pathological myocardial hypertrophy. While previous studies have shown that 11β-HSD1 knockout mice have a better post-MI heart function [20], [21], this is the first study showing the potential utility of pharmacological inhibition of local glucocorticoid activation, in general and of 11β-HSD1 inhibition, specifically, to efficiently reverse pathological hypertrophy. Our findings that this key enzyme in glucocorticoid activation is strongly upregulated in the course of hypertrophy and, in turn, down regulated upon resolution of hypertrophy via neovascularization has provided incentive to examine a causative role for 11β-HSD1 in the process and for its pharmacologic inhibition. Interestingly, 11β-HSD1 inhibition also led to its reduced expression (Fig S5) suggesting a direct or indirect positive regulation of 11β-HSD1 and intensifying the effect of its inhibition. At the molecular level, 11β-HSD1inhibition resulted in a myosin heavy chain isoform shift from the fast-contracting MyHCα to the slow contracting MyHCβ. As marked by reversing the upregulated expression of BNP and periostin (a TGFβ target gene), 11β-HSD1inhibition also acted to relieve wall stress. It is assumed that the failure to restore normal cardiac function via 11β-HSD1inhibition in this system in face of ongoing VEGF blockade is due to persistent ischemia enforced by continued inhibition of angiogenesis. Thus, combining pro-angiogenic therapy with 11β-HSD1inhibition might have an additive effect.

## Supporting Information

Figure S1 (TIF)

Figure S2 (TIF)

Figure S3 (TIF)

Figure S4 (TIF)

Figure S5 (TIF)
