# Supplementary figures and images for: A Transgenic Platform for Testing Drugs Intended for Reversal of Cardiac Remodeling Identifies a Novel 11βHSD1 Inhibitor Rescuing Hypertrophy Independently of Re-Vascularization
Source: PLoS One. 2014 Mar 25;9(3):e92869. doi: 10.1371/journal.pone.0092869 (PMC3965501; doi:10.1371/journal.pone.0092869)

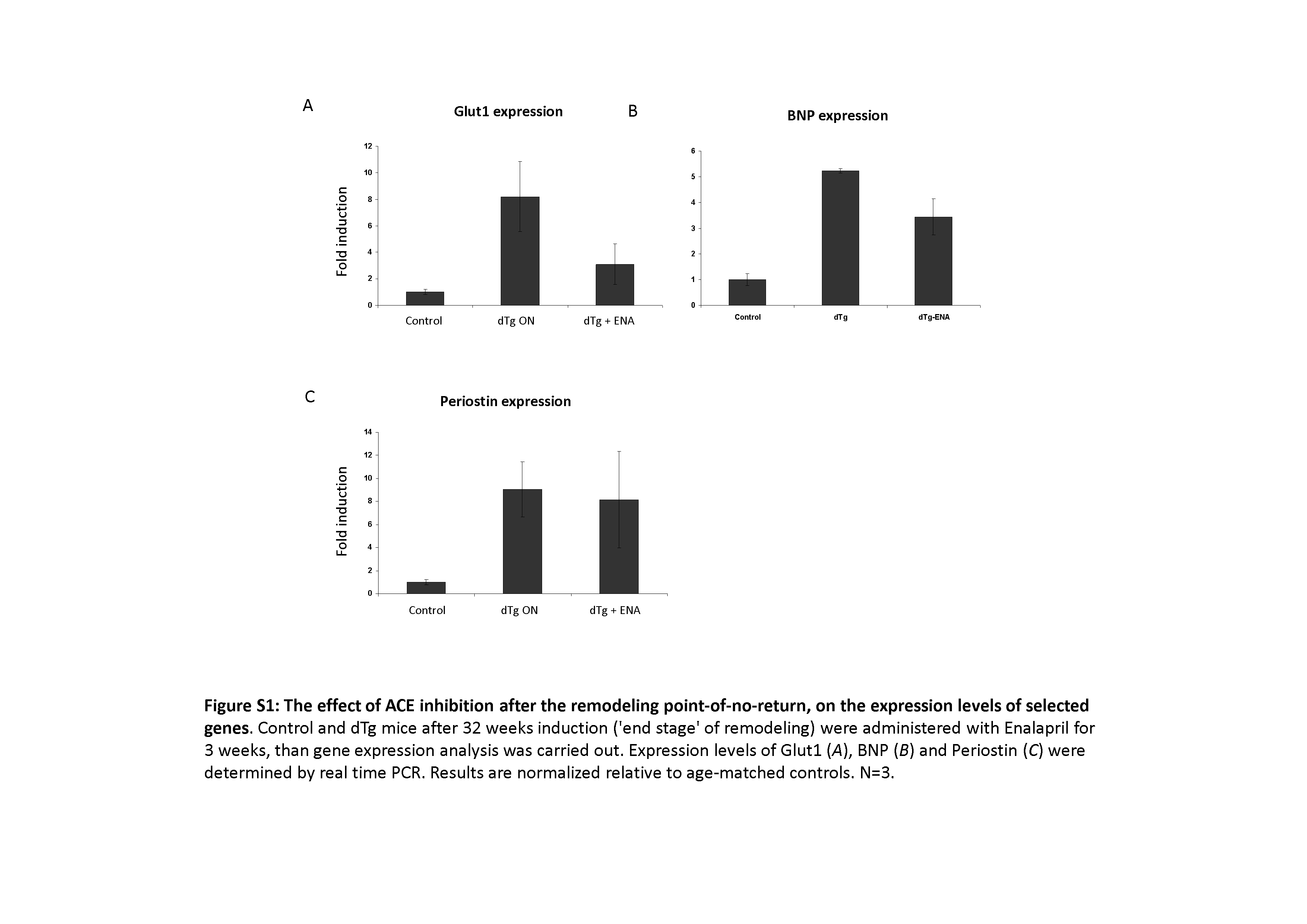

Supplement: Figure S1 — (TIF) [file pone.0092869.s001.tif]

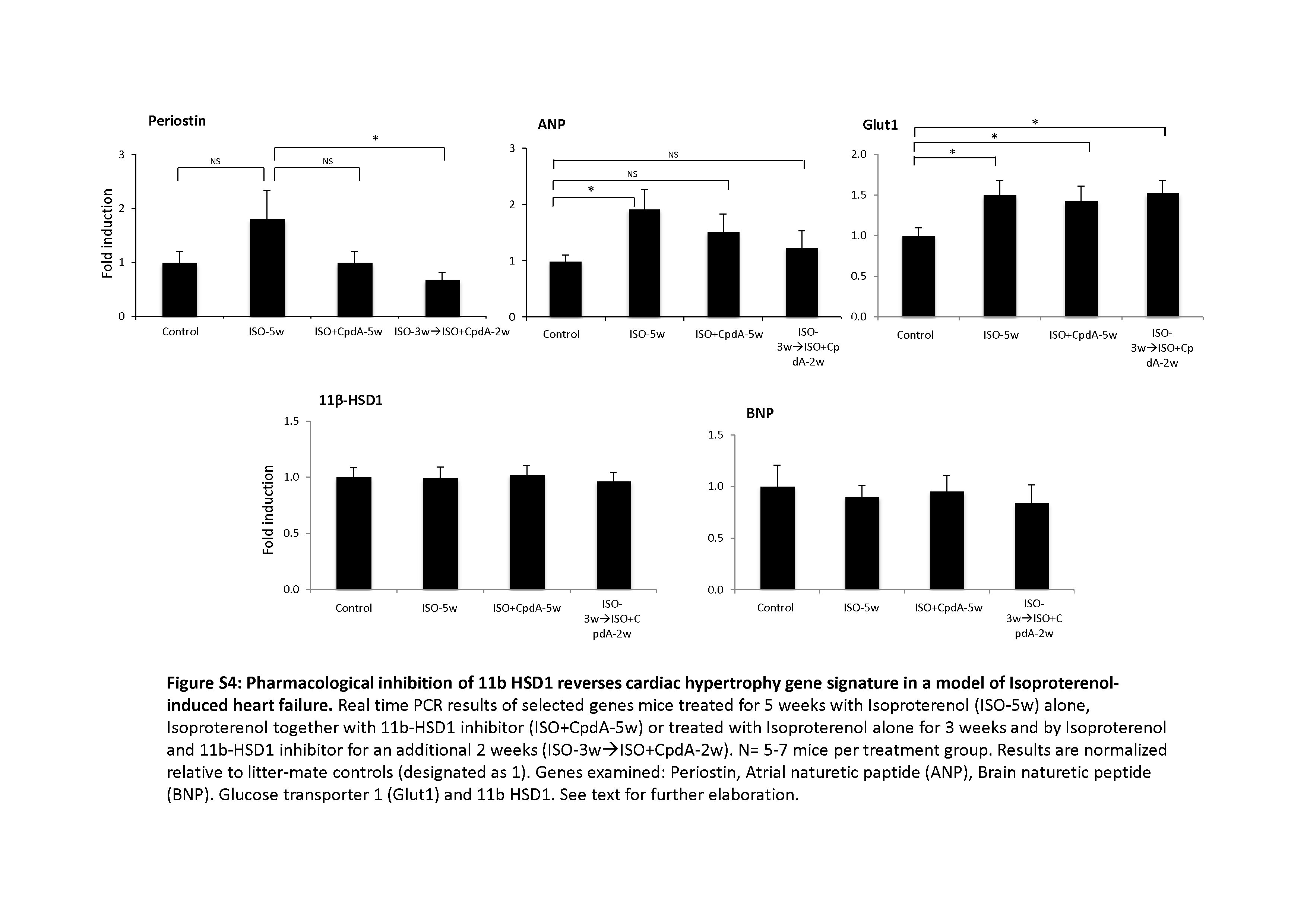

Supplement: Figure S4 — (TIF) [file pone.0092869.s004.tif]

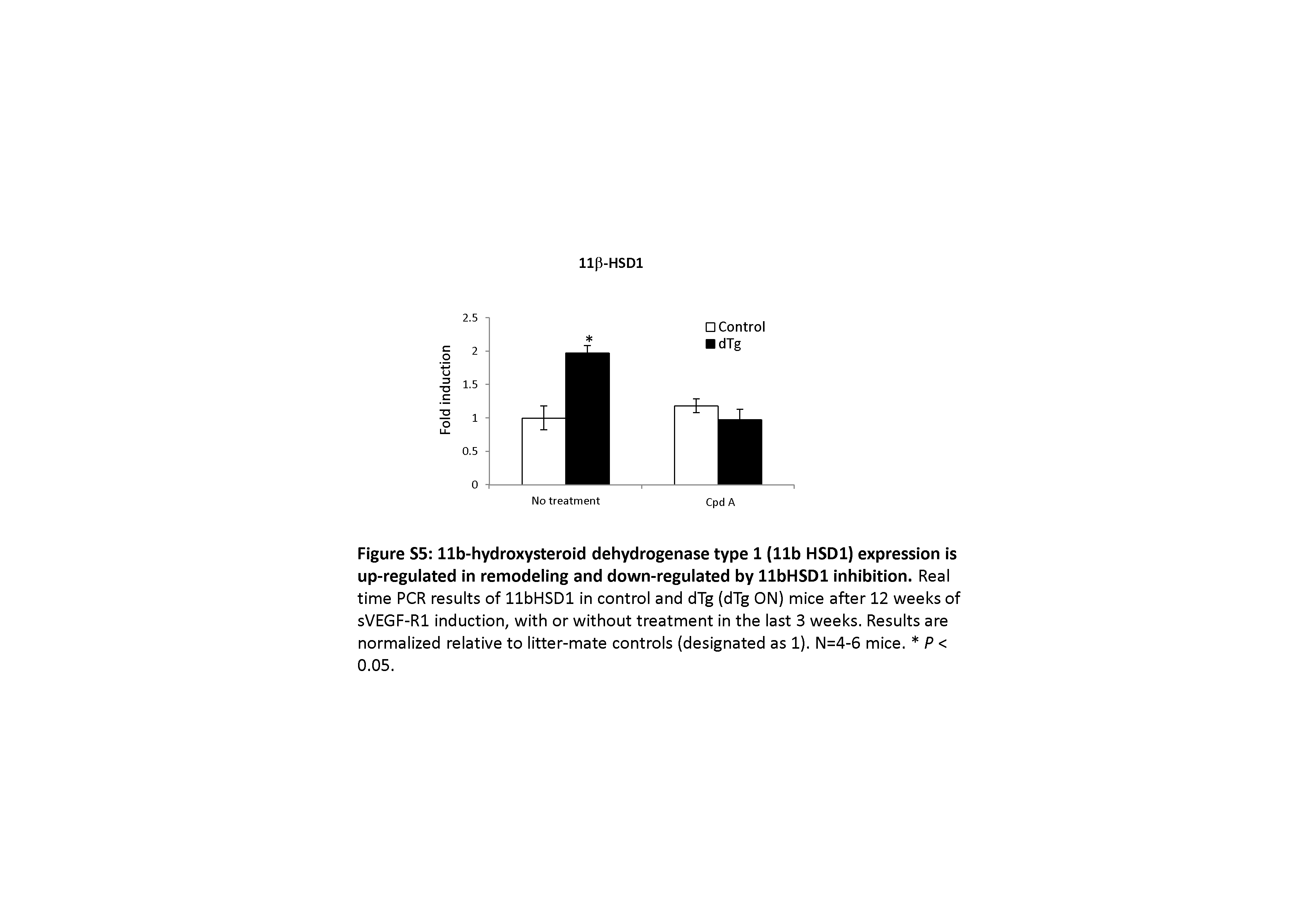

Supplement: Figure S5 — (TIF) [file pone.0092869.s005.tif]
